# Artificial intelligence, the magic circle, and CBT: a Wizard-of-Oz framework for adjunctive treatment

**DOI:** 10.3389/fpsyt.2026.1941336

**Published:** 2026-09-23

**Authors:** Zhong Zheng, Luosi Wang, Xiang Cheng, Xuexing Luo

**Affiliations:** 1 College of Arts and Design, Lingnan Normal University, Zhanjiang, Guangdong, China; 2 School of Art and Architecture Design, Zhuhai University of Science and Technology, Zhuhai, China; 3 School of Computer Science and Intelligence Education, Lingnan Normal University, Zhanjiang, Guangdong, China

**Keywords:** artificial intelligence (AI), asynchronous Wizard of Oz (AWoZ), board games, cognitive behavioral therapy (CBT), cognitive defusion, magic circle, recontextualization

## Abstract

Cognitive behavioral therapy (CBT) is a cornerstone of evidence-based psychological treatment, but its implementation continues to be limited by poor patient adherence and shortages of clinical resources. At the same time, most applications of artificial intelligence (AI) and games in mental health have focused on direct intervention, although the evidence base remains underdeveloped and AI has important limitations in diagnosis and treatment decisions. In response, this article proposes a Wizard-of-Oz (WoZ) framework for adjunctive treatment that positions AI and board games as tools that support conventional CBT rather than replace it. The framework draws on the mechanism of recontextualization in acceptance and commitment therapy (ACT). By introducing an alternative functional context, recontextualization reduces the control that verbal relations exert over behavior and helps patients shift from cognitive fusion (“I am my thoughts”) to an observer perspective (“I am noticing my thoughts”). The physical interaction and rule system of a board game create an embodied magic circle in which cognitive defusion and cognitive preparation can be practiced in a low-pressure, reversible, and symbolic environment. The AI module remains an information curator and prudent advisor: it organizes structured records and produces descriptive summaries of game behavior and experience logs to assist, rather than determine, the therapist’s clinical judgment. In the proposed implementation, physical board actions are entered through a constrained digital companion interface; the architecture does not assume RFID, sensor-augmented boards, computer vision, or other automatic sensing. The framework uses an asynchronous Wizard-in-the-Loop design. The therapist does not need to intervene during play but reviews structured, AI-assisted summaries in the background and remains outside the patient’s immediate view. The digital companion provides rule prompts and a controlled form for recording board choices, dice outcomes, piece movements, and voluntary reflections. This arrangement preserves human clinical judgment while reducing patient defensiveness. The game design minimizes external rewards such as points and leaderboards and instead emphasizes the deeper experiences created by feedback and challenge. It also uses three types of metaphor—maps, role-play, and monsters—to integrate embodied cognition with the learning of CBT skills. At the ethical level, the framework addresses concerns about emotional dependence, cultural bias, privacy, and data security through prior informed consent, data minimization, de-identified presentation, and patient control over data. Its main contribution is a cautious and practical design strategy that does not depend on the still-debated effectiveness of direct AI intervention. Instead, it uses AI and games to reduce peripheral barriers and prepare patients for therapist-led CBT, with the aim of improving adherence, making better use of limited resources, and reducing treatment resistance.

## 1 Introduction

Cognitive behavioral therapy (CBT) is among the most widely used and well-supported psychological interventions in psychiatry and clinical psychology. Its central approach is to identify, evaluate, and modify unhelpful cognitions, such as negative automatic thoughts, and thereby change maladaptive behavior (1, 2). However, conventional face-to-face CBT has long faced two major barriers in clinical practice. First, patient adherence is often poor. CBT relies on structured behavioral experiments and regular homework. This rational, repetitive, and closely self-monitored process can lead to resistance and contribute to high dropout rates. Second, specialist clinical resources are severely limited. Training qualified therapists is costly and time-consuming, and the available workforce is far from sufficient to meet the growing global need for mental health care (3, 4).

Game-based therapy has therefore received increasing academic attention. Among different forms of game intervention, board games may offer distinctive clinical value because they provide physical interaction rather than screen-based virtual interaction alone. From the perspective of embodied cognition, tactile activities such as handling a board, rolling dice, and moving pieces can engage grounded cognitive processes (5, 6). They may also produce stronger emotional engagement and a greater sense of presence than visual stimulation alone. More importantly, the rules of a board game create a natural magic circle in physical space. Within the game, players temporarily accept a separate set of rules, roles, and meanings, allowing them to try actions that may be difficult in everyday life within a low-pressure, reversible, and symbolic setting (7). In psychotherapy, the key value of the Magic Circle lies not only in its aim to provide a safe, fun, and detached intermediary space, but also in its intention to create a low-pressure, supportive learning environment. Patients can test ways of thinking with little risk and rehearse core CBT concepts, such as recognizing cognitive distortions and labeling emotions. This implicit learning and cognitive preparation may support later clinical interviews and more intensive treatment (8).

Within this setting, the direct and serious relationship between therapist and patient, which is often shaped by differences in authority, is temporarily supplemented by another form of interaction. Rather than responding only to the therapist’s questions and explanations, the patient acts within a system made up of game rules, a physical board, roles, narrative events, and AI support. This system is the magic circle. As an enriched setting that supplements and buffers direct clinical intervention, it may reduce shame, defensiveness, and fear of evaluation (9).

Current combinations of AI and games in health care remain largely at the feasibility-testing stage, and high-quality randomized controlled trials have not yet established their effectiveness as direct interventions for mental disorders. Because the theoretical and clinical evidence is still developing, assigning a central therapeutic role to games, whether or not they include AI, involves substantial uncertainty (10, 11).

In their framework for human-centered AI design, Thieme et al. argued that current AI is not sufficiently mature to assume independent responsibility for diagnosis or intervention in psychotherapy. A more practical approach is to use AI as a partner that supports clinical decision-making (12).

A more cautious approach may therefore be valuable. Mauriello et al.’s study of Popbots showed that a conversational agent based on predefined scripts, without complex semantic understanding, could still reduce depressive symptoms. Users also appeared to divide tasks naturally between technical tools and human experts: they were more willing to assign low-complexity activities, such as daily reflection, emotion labeling, and cognitive reminders, to a machine (13).

Accordingly, this article proposes a conceptual architecture for game-assisted psychotherapy. The board game does not address deep clinical pathology directly. Instead, it provides cognitive preparation and serves as an information bridge, reducing the preliminary cognitive and administrative demands placed on the treating clinician.

This adjunct is not appropriate for every patient. It is limited to patients for whom CBT is clinically indicated and whose cognitive and behavioral abilities are sufficient to manage the demands of a board game. A clinician should determine suitability after an appropriate assessment.

In the early development of complex behavioral interventions, the WoZ paradigm has been used to evaluate interaction design and user acceptance before a system is fully implemented (13). The present framework applies this approach by redefining the therapist’s “behind-the-scenes control” as a mechanism for continuous clinical observation and data annotation: the therapist reviews the “behavior logs” and “experience logs” generated through the digital companion, as well as the analytical reports provided by the AI, without directly intervening in the game process. The therapist does not monitor the background data in real time; this is an AWoZ mechanism. The label is used deliberately: AWoZ describes an interaction-design and evaluation configuration in which the patient encounters an apparently automated operational interface while a hidden human reviews information after play. It does not imply that the AI autonomously makes clinical decisions.

### 1.1 AWoZ should be distinguished from related human-oversight labels

In an asynchronous human-in-the-loop (AHITL) system, the human’s intervention is part of the system’s active processing and may alter the output before it is delivered. In a human-on-the-loop decision support system (DSS), an automated model typically produces recommendations or decisions while a human supervises, monitors, or intervenes in the operational loop. In contrast, the present architecture uses a nonclinical digital companion for rule delivery and data capture; the therapist is hidden during play and reviews records after the session, while the AI only organizes information. Thus, AWoZ names the behind-the-scenes, asynchronous human mediation of the interaction—not an autonomous clinical DSS. If implemented in a later stage, the therapist’s retrospective review would remain a separate clinical oversight layer.

The central question of this article is as follows: How can AI and board games create AWoZ information architecture and an embodied magic circle that reduce cognitive and emotional barriers between therapist and patient without directly treating psychopathology, while providing practical support for the limitations of conventional CBT in adherence, resource availability, personalization, and treatment resistance?

## 2 Magic-circle architecture: AWoZ-based context for cognitive defusion

Recent work has challenged the traditional view that cognitive restructuring in CBT and cognitive defusion in ACT are entirely opposed or mutually exclusive. In practice, patients may combine the two strategies flexibly when coping with distress rather than relying on only one approach (14). Both strategies have been associated with improvements in positive emotion and progress toward personal values, whereas defusion may have more specific benefits for reducing rumination and negative emotion. Momentary restructuring and momentary defusion are also significantly associated within the same time period, suggesting that an increase in one strategy at a given moment is often accompanied by an increase in the other (15).

This framework draws on ACT as a practical resource rather than adopting it as the primary treatment model. It does not replace conventional CBT with ACT. Instead, it incorporates ACT’s emphasis on context and psychological distance in behavior change (16) as an adjunctive strategy. The ACT-informed game and AI components are limited to creating a safe space for trial and error and making implicit thoughts easier to observe. Their purpose is to reduce psychological defensiveness and cognitive rigidity so that the patient is better prepared for therapist-led cognitive restructuring. Changes to core beliefs remain within the professional therapist’s clinical judgment and are guided throughout by the conventional CBT framework.

### 2.1 Recontextualization within the magic circle

Assaz et al. identified recontextualization as one of the central processes of cognitive defusion in ACT. It introduces an alternative functional context, such as spatial distance or a narrative label, that reduces the control of verbal relations over behavior without changing the content of the thought itself. In this process, a thought or experience is shifted from an immersed, first-person perspective to a more distant observer perspective (16).

Eifert and Heffner used an experiential metaphor in which participants handled a Chinese finger trap and experienced how struggling could make the trap tighten (17). This method informs the present framework: the board game itself is designed as a large, interactive metaphor. Unlike verbal instruction alone, physical actions such as rolling dice, moving pieces, and turning over game components create a structured space with rules, feedback, and consequences. Each decision produces clear and immediate feedback. Through this feedback loop, abstract cognitive strategies, including weighing costs, shifting perspective, and tolerating uncertainty, are experienced through action rather than understood only at an intellectual level. The game does not prescribe a particular conclusion. Its structure instead allows players to try new cognitive and behavioral patterns repeatedly in a low-risk environment, creating a learning experience similar to an experiential metaphor.

Recontextualization is the central design mechanism of the board-game framework. It creates a safe space for trial and error outside the patient’s usual verbal context. Within this space, the magic circle, the game mechanics and AI support jointly make implicit thoughts easier to observe. First, the physical board externalizes cognitive conflict symbolically by creating spatial distance and turning it into a concrete game event. Second, the AI presents implicit responses as readable text through neutral logging and pattern labels. Together, these processes help the patient shift from fusion (“I am my thoughts”) to an observer perspective (“I am noticing my thoughts”), creating a less defensive state in preparation for subsequent therapist-led cognitive restructuring (16).

Flexible difficulty options create a flexible gameplay pace, giving players ample room to tackle challenges at their own pace. Based on the theory of flow, these levels allow patients to independently balance challenge and skill, reducing resistance to activities that might otherwise be experienced as clinical tasks. As patients overcome challenges and collect game items, they can gradually develop a sense of achievement and self-efficacy, which may support continued engagement (18, 19).

### 2.2 The therapist as an asynchronous wizard in the loop

Conventional CBT often requires frequent, direct, face-to-face contact between therapist and patient, creating a substantial workload. To reduce demands on specialists in an online CBT program, Zainal et al. assigned nondiagnostic tasks, such as writing and sending conversational messages, to human coaches and assigned retrospective quality monitoring to AI. However, the best AI classifier achieved an area under the curve of only 76.06%, well below the level required for clinical decisions. Approximately one quarter of its classifications could differ from those of human experts, potentially increasing the need for review (20). These limitations suggest that, under current technical conditions, placing AI in any decision-making role carries substantial risk.

The core essence of traditional WoZ is that users believe they are interacting with an autonomous system, although one or more hidden humans actually observe, control, or manage the interaction. In the present framework, the therapist is the hidden Wizard. The therapist does not appear in the game interface or control play in real time but remains a background observer and the final clinical decision-maker outside the magic circle. Here, “hidden” refers to the therapist’s absence from the immediate game interface, not concealment from informed consent: patients are told that the therapist will receive the resulting records.

During the session, the architecture is presented through a digital companion that includes a rule-based virtual assistant. The patient sits at a physical game board and interacts with the assistant through a computer program or mobile application. The assistant explains the rules, prompts the player about turn progression, and provides immediate operational feedback, giving the patient a continuous sense that the system is present. Its role, however, is strictly limited to process management, information delivery, and structured data entry. Its dialogue concerns the game rules and does not include clinical judgments or psychological guidance. The patient is informed that the board game forms part of treatment and that the therapist will receive information from the game in the background. Because the therapist does not participate in the game interaction, however, the patient does not experience the therapist as immediately present. This separation is the central WoZ feature of the architecture. In this conceptual version, the physical–digital bridge is manual rather than sensor-based: the player records each relevant board choice, dice outcome, piece movement, and optional strategy selection in a constrained app interface. The app timestamps these entries and checks them against the current game state, but it does not claim to have sensed the physical action itself.

The difference between traditional WoZ and the AWoZ in this study is the timing and visibility of human involvement. The therapist does not need to be present or respond in real time while the patient plays. The virtual assistant manages the session, and the patient completes the board-game activities independently. After the game, or between clinical appointments, the therapist reviews two records in the background. The structured interaction log contains app-recorded entries for board choices, strategy selections, dice outcomes, piece movements, and response times; because the physical actions are manually entered, these fields are treated as user-entered records rather than automatically sensed objective measurements. The experience log contains the patient’s own reflections, emotion labels, and account of learning. The two records remain distinguishable by provenance, so the therapist can see which information was entered by the patient and which was generated by the digital companion. Through this process, the therapist remains offline during play and re-enters the loop afterward by reviewing the logs.

This asynchronous arrangement also keeps the therapist out of sight during play, so the patient does not feel that someone is watching. The therapist’s lack of immediate visibility may aim to reduce defensive responses often seen in clinical interviews, such as impression management and fear of negative evaluation. The patient may therefore be more likely to show natural behavioral tendencies and cognitive patterns in the low-pressure, reversible, and symbolic game environment.

To support the safety and appropriate use of this AWoZ architecture, the design includes safeguards at three levels.

First, the content supports cognitive defusion. The game’s narratives, cases, and discussion prompts refer to external subjects: the patient is playing a game, discussing knowledge, or analyzing another person’s case rather than directly confronting personal pathology or trauma. This “depersonalized” design is intended to help patients psychologically create a safe distance from the distressing content, thereby reducing the risk of triggering strong negative emotional reactions.

Second, the presentation remains gentle and controlled. All game prompts and virtual-assistant dialogue come from a professionally reviewed content library and are not generated independently by AI. Difficulty is unrelated to rewards and increases only when the patient’s cognitive load is manageable and the patient clearly wishes to proceed. Difficulty can also be reduced at any time.

Third, patient screening establishes a threshold for appropriate use. The adjunctive system is not suitable for all patients. It is limited to patients for whom CBT is clinically indicated and whose cognitive and behavioral abilities are sufficient for the basic demands of board-game play. It is not appropriate for patients with severe cognitive impairment, acute psychotic symptoms, or a high risk of suicide. The treating clinician should determine suitability after a comprehensive assessment.

At the conceptual stage, the AI module is specified as a hybrid, auditable, non-diagnostic text-processing pipeline rather than as an autonomous generative therapist. First, a schema-validation and time-alignment layer checks the structured interaction records and separates app-entered events from voluntary reflection text. Second, a clinician-curated CBT and emotion lexicon, with lemmatization and controlled-vocabulary normalization, supports keyword and emotion-term extraction; a transparent TF–IDF ranking may be used to identify salient terms, but it is not interpreted as a clinical score. Third, emotion-related information is summarized as counts or frequencies of words and patient-selected labels in the reflection log. These features describe the record and do not infer hidden affect, diagnosis, or risk. Fourth, entries are linked to CBT materials through rule-based matching against indexed metadata such as cognitive distortion, skill, and difficulty level. Embedding-based similarity or a transformer classifier is not assumed in the present design; if evaluated in future work, it would require separate validation and would remain restricted to descriptive retrieval. Finally, a template-based summary presents source entries, extracted terms, and provenance flags to the therapist. No large language model is required for the proposed baseline, and any future generative summarizer would need to be retrieval-constrained, auditable, and subject to human review. When interacting with patients, the AI provides procedural and cautious recommendations. Based on interaction data, it offers non-mandatory, optional prompts to patients (such as suggestions for adjusting difficulty levels)—but the authority to make any adjustments always rests with the patient; the AI does not perform any automatic adjustments.

This provenance rule is intentional: a manually entered board event is a report of what the player recorded, not evidence that the software directly observed the physical board. A future smart-tangible implementation could automate some fields through RFID, board sensors, or computer vision, but that would be a separate technical extension requiring new consent, validation, and privacy analysis.

In summary, the therapist observes asynchronously from outside the magic circle, remains outside the patient’s view within the AWoZ architecture, uses AI-supported information organization, and makes all final decisions during clinical consultations. This design reduces peripheral work involved in collecting and organizing information, uses the therapist’s lack of immediate visibility to reduce patient defensiveness, and avoids placing AI in a decision-making role for which it is not yet suited.

## 3 Game elements and mechanisms within the magic circle

### 3.1 Strategy for selecting game elements

A board game used to support CBT should not follow all the design principles of commercial entertainment games, because addictive patterns of use and distress caused by failure must be minimized. This is reflected first in the selection of game elements. Common elements include points, leaderboards, levels, feedback, and challenges (21).

Game elements can be divided into surface structures and deeper experiences (1). External evaluation indicators, or surface structures, include points, leaderboards, levels, badges and rewards, and unlockable content. These forms of operant conditioning provide immediate external feedback and social comparison, but continued reliance on them can shift attention toward playing for points. Interest may decline quickly after the novelty wears off. In the present framework, these surface elements should be minimized and may be omitted (2). The internal core of the game, or the deeper experience, includes feedback and challenge, as well as quests, customization, and avatars. These features relate directly to psychological needs. A well-matched challenge can support competence and the experience of flow. Feedback, including progress cues, narrative responses, expanded content or forms of participation, and links to homework, can support autonomy and relatedness by helping players understand the results of their actions. The deeper experience is therefore central to the proposed design.

### 3.2 Metaphors in the game

The use of game metaphors in psychotherapy is a well-established area of academic study, with a growing theoretical and empirical literature (23, p. 21; 24). Common metaphors in games include maps, role-play, and task-based levels.

In the present framework, the map serves both embodied and symbolic cognitive functions and brings them together. At the level of player action, it uses four paired behavioral and psychological processes: exploration and immersion, physical manipulation and kinesthetic experience, role-play and cognitive defusion, and reflective writing and cognitive restructuring. At the symbolic level, different areas represent five types of space: a starting point, a skill-learning area, challenge levels, a rest area, and an endpoint that confirms growth. Together, these functions make the map more than a passive background. It becomes an embodied game space through which the player can move, perceive, and choose.

More specifically, the map as a whole provides space for exploration and immersion, with the starting point serving as the entry into that experience. The game levels provide settings for physical manipulation and kinesthetic activity. The learning and challenge areas allow role-play and cognitive defusion, whereas the rest area regulates the pace and gives players an opportunity to write reflections in preparation for cognitive restructuring. The endpoint provides an important form of positive feedback.

The role-play component draws on 12 common verbal intervention strategies used in conventional CBT (22). Three of these strategies - evidence gathering, consequential analysis, and generating alternative explanations - can involve reconsidering one’s experience from an external perspective. This feature may represent a practical point of contact between CBT and ACT, despite their philosophical differences. In the present framework, these strategies inform three forms of role-play, each based on another person’s perspective at a different point in time. The evidence reviewer examines past experience to test the accuracy of a current belief. The observer adopting another person’s perspective uses a present-focused, empathic viewpoint to step outside a cycle of self-attribution. The future reviewer creates temporal distance and uses a longer-term perspective to reduce the immediate impact of emotion. Together, the three roles cover past, present, and future perspectives and provide an operational framework for cognitive defusion. The framework uses ACT-related perspective shifting and distancing to support cognitive change, but it does not end with acceptance alone. Instead, the shift in temporal and spatial perspective provides richer and more distanced material for therapist-led cognitive restructuring in CBT. Defusion therefore serves restructuring as a practical method rather than replacing it as the final aim.

The third game metaphor is the monster encountered during game levels. Monsters represent common forms of distorted thinking. As players progress through the board game, they select different character perspectives, confront the monsters, and pass through learning areas that introduce CBT skills. They can thus practice basic cognitive restructuring skills from a safe distance.

### 3.3 Coordinating play and learning

The game includes two knowledge bases to support learning about cognitive restructuring. The Cognitive Distortion Knowledge Base presents common forms of cognitive distortion in CBT and their identifying features. A second knowledge base presents 12 common verbal intervention strategies and explains core cognitive restructuring techniques within CBT (22). Importantly, the cognitive restructuring knowledge base provides educational content rather than practical training. Players learn about the principles and appropriate contexts of different behavioral strategies through reading and cases, but they are not required to perform these techniques in the game. The game provides cognitive preparation only. All behavioral interventions remain under the therapist’s clinical judgment and guidance.

## 4 Ethical Considerations and future directions

The first major risk of game-based therapy is excessive emotional dependence and the appearance of companionship. People with mental disorders may form strong emotional projections and human-like attachments to AI systems that respond with apparent empathy. An AI system that is always available and consistently accommodating may become a preferred source of comfort during periods of distress. This type of frictionless interaction may displace human relationships and worsen existing symptoms. AI companionship may also lead to grief over a relationship that felt real and to unhealthy emotional dependence. The system should therefore avoid presenting the AI as the only source of understanding and should guide the player back to real interaction under the therapist’s supervision. The purpose of the magic circle is not long-term immersion but safe practice that can be transferred to everyday life (23, 24).

The second concern is cultural bias. AI systems for mental health have been developed mainly from data on Western, educated, industrialized, rich, and democratic (WEIRD) populations (25). In psychiatry, one study found that an AI model used to predict aggressive incidents produced significantly higher false-positive rates for Black and Middle Eastern patients, so these groups were disproportionately classified as high risk (26). If fairness is not included in system design, clinical use of AI may cause distress and reduce trust. Culture should therefore be treated as a core design requirement throughout the AI development process rather than added afterward (27). In the present architecture, the AI module does not make diagnoses or culturally sensitive judgments; it only structures behavioral logs. This restriction may reduce the amplification of cultural bias in algorithmic outputs.

The third concern is privacy and data security, particularly the tension between transparency and the implicit delivery of treatment-related content. Embedding therapeutic techniques in game mechanics does not necessarily involve deceiving patients, but its ethical acceptability depends on clear, prior informed consent. Patients should understand that (1) the system supports a mental health intervention, (2) the digital companion records manually entered game events and voluntary experience-log entries, (3) AI uses those records only to produce descriptive summaries and optional procedural prompts, and (4) the therapist will not participate in game interaction but will observe the resulting information in the background. Previous work has called for AI governance that provides separate, explicit opt-in consent and gives patients a leading role in decisions about their data. In the present framework, affective computing refers narrowly to text-based affect-related feature extraction from voluntary reflection logs and patient-selected emotion labels. Response latency, number of edits, and interaction pace may be retained as behavioral or engagement proxies, but they are not physiological or affective biomarkers and are not used to infer a hidden emotional state. The system does not collect physiological, facial, or voice data, and it does not use computer vision or smart-board sensing in the proposed implementation. AI-generated summaries are presented to the therapist in de-identified form, with the original logs available for review, and patients can access and delete their data. Transparency is therefore both an ethical requirement and a basis for trust in the treatment framework (28, 29).

The framework is currently at the conceptual design stage and requires a systematic validation process before clinical deployment. Future research could proceed along the following pathway. First, co-design with patients and clinicians should be used to refine the game mechanics and user interface, ensuring their acceptability and clinical relevance. Next, usability testing and technical evaluation should assess the system’s stability, ease of use, and completeness of its log data. On this basis, small-scale safety assessments and feasibility studies should be conducted to provide preliminary evidence of the framework’s safety and practicality in the target population. Finally, if the results of these stages are satisfactory, randomized controlled trials (RCTs) should be conducted to rigorously evaluate the framework’s clinical benefits as an adjunctive tool. This stepwise validation pathway is intended to ensure that the tool has sufficient evidence of safety, usability, and preliminary effectiveness before it is introduced into clinical practice.

## Data Availability

The original contributions presented in the study are included in the article/supplementary material. Further inquiries can be directed to the corresponding author.
